# A novel signal channel attention network for multi-modal emotion recognition

**DOI:** 10.3389/fnbot.2024.1442080

**Published:** 2024-09-11

**Authors:** Ziang Du, Xia Ye, Pujie Zhao

**Affiliations:** Xi'an Research Institute of High-Tech, Xi'an, Shaanxi, China

**Keywords:** hypercomplex neural networks, physiological signals, attention fusion module, multi-modal fusion, emotion recognition

## Abstract

Physiological signal recognition is crucial in emotion recognition, and recent advancements in multi-modal fusion have enabled the integration of various physiological signals for improved recognition tasks. However, current models for emotion recognition with hyper complex multi-modal signals face limitations due to fusion methods and insufficient attention mechanisms, preventing further enhancement in classification performance. To address these challenges, we propose a new model framework named Signal Channel Attention Network (SCA-Net), which comprises three main components: an encoder, an attention fusion module, and a decoder. In the attention fusion module, we developed five types of attention mechanisms inspired by existing research and performed comparative experiments using the public dataset MAHNOB-HCI. All of these experiments demonstrate the effectiveness of the attention module we addressed for our baseline model in improving both accuracy and F1 score metrics. We also conducted ablation experiments within the most effective attention fusion module to verify the benefits of multi-modal fusion. Additionally, we adjusted the training process for different attention fusion modules by employing varying early stopping parameters to prevent model overfitting.

## 1 Introduction

Multi-modal signal recognition is a critical area of research within multi-modal fusion, encompassing fields such as speech signal recognition, physiological signal recognition, and radar signal recognition. The primary task is to classify multi-modal signals from the same individual, as individual signals alone often fail to capture the comprehensive features required for study. Thus, developing multi-modal signal classification models is crucial for a deeper understanding of these signals.

Using physiological signals for emotion recognition is a significant approach to studying human emotions. Since expressions and speech can conceal emotions and behavioral responses can suppress abnormal emotions, the advent of non-invasive and affordable wearable devices has propelled deep learning-based physiological emotion recognition into a prominent research area. Common physiological signals used include electroencephalogram (EEG), electrocardiogram (ECG), galvanic skin response (GSR), and eye data, etc. In the past, emotion recognition tasks frequently relied on data involving facial expressions or speech signals. Unlike these outward expressions, EEG signals offer a direct window into the brain's physiological activity, making them less prone to artifacts or manipulation and thus providing a more authentic and unbiased view of one's emotional state. ECG signals, on the other hand, directly reflect the heart's activity, which is closely tied to emotions, and are adept at capturing physiological responses to emotional shifts. ECG typically boasts a higher signal-to-noise ratio than EEG and is more easily obtained and analyzed non-invasively. GSR is particularly sensitive to an individual's emotional arousal, such as nervousness or excitement, often marked by changes in skin conductance. Eye movement signals are closely linked to attention and interest, with emotional states deduced from the duration and direction of gaze. While each of these modalities offers unique benefits for emotion recognition, there are relatively few studies that have harnessed all four signals in tandem for this purpose. These signals serve as inputs for deep learning models in emotion classification.

With the rise of deep learning, Advanced neural networks with attention modules have also proliferated in recent years. Kalman filters can be combined with residual neural networks to obtain even better neural networks (Yang et al., 2023a). A novel deep saliency-aware bi-embedded attention network (SAD-Net) for non-periodic multivariate time series prediction and has demonstrated high performance on correlated datasets (Li et al., 2023). Multi-layer fully connected networks and lightweight graph-convolutional networks can be fused into a dual-stream graph-convolutional network fusing potential features, who can solve the key problem of linear properties and the limitations of implicitly encoded cooperative QoS signal (Bi et al., 2023). Concurrently, numerous physiological signal classification models have been developed annually. Among these, multi-modal hyper complex neural network models show great potential and have achieved notable success in physiological signal classification. These models utilize multi-modal physiological signals from various emotional states, surpassing previous methods focused solely on EEG signals. Past research has predominantly remained single-modal and lacked the capability to extract comprehensive data features using deep learning models. To address this challenge, the authors proposed a hyper complex neural network model (Lopez et al., 2023).

In current research on multi-modal neural networks, the varying significance of different information types in determining data features remains unresolved, limiting the training potential of these models. Proper deep learning neural network models can adjust the weights of each modality to optimal values, and incorporating attention mechanisms can enhance model performance. However, due to inadequate attention mechanisms, hyper complex neural networks have not yet achieved their expected performance. Therefore, increasing attention mechanisms will help neural networks better capture key information, thereby improving model effectiveness.

**Figure 1:**
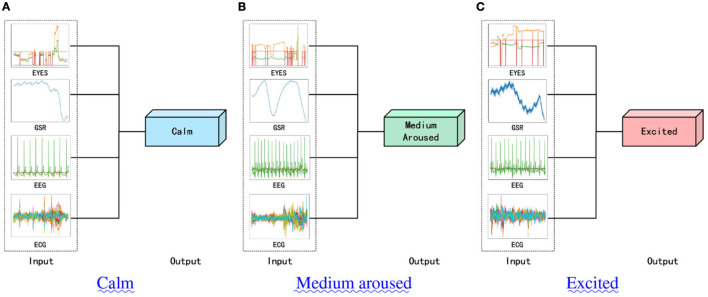
The summary of the model we designed. **(A)** shows a sample of data predicted as “Clam,” **(B)** shows a sample of data predicted as “Medium surrounded,” and **(C)** shows a sample of data predicted as “Excited.”

To this end, we have enhanced the existing multi-modal signal classification model, specifically the multi-modal hypercomplex neural network model, by incorporating five newly designed attention modules. This has led to the development of five improved models that outperform previous hypercomplex multi-modal signal models in the realm of physiological signal research. Crucially, we preserved the original model's innovative aspects. Through multiple model comparison experiments and ablation studies using the publicly available benchmark dataset MAHNOB-HCI, we verified the efficacy of our approach. Our model demonstrated significant performance improvements. Figure 1 summarizes the tasks our designed network will undertake, with the three images illustrating the different classification samples corresponding to our final three classification tasks. Additionally, this study identified shortcomings in the data processing and conclusions of previous research, which we will address in detail in the paper.

Our contributions can be summarized as follows:

- We have designed a novel framework based on previous research, named SCA-Net, which achieves higher accuracy and better prediction balance in the field of multi-modal physiological signal recognition.
- We discovered that improvements in multi-modal physiological signal processing models can be attained through two types of attention methods: channel attention and self-attention. Incorporating both types of attention resulted in varying degrees of enhancement in the model's performance.
- We conducted comparative experiments on five models using the publicly available dataset MAHNOB-HCI, both with and without data augmentation. Additionally, we performed ablation experiments on the best-performing models to validate the effectiveness of our approach.

## 2 Related work

In recent years, the advantages of deep learning have become increasingly evident, sparking a renewed enthusiasm for emotion recognition research. Extensive studies have already been conducted across various domains, including natural language processing, computer vision, and signal processing. Broadly speaking, these studies fall into two categories: emotion recognition based on a single modality and emotion recognition based on multiple modalities.

### 2.1 Emotional recognition under single modality

In the past many studies, and there have been many methods that can be used for emotion recognition under a single mode (Rayatdoost and Soleymani, 2018; Wang et al., 2019; Du et al., 2020; Maeng et al., 2020), but when these neural network frameworks carry out emotion recognition tasks, they excessively rely on extracted features, such as power spectral density (PSD) and differential entropy (DE), which ignore the ability of neural networks to recognize and extract features. Among the numerous available data, facial images can be used as an auxiliary means to preliminarily recognize emotions (Tao et al., 2020), and the speech signals received by sensors can also be used as a means of emotion recognition (Sajjad et al., 2020; Shen et al., 2024). ECG signals also have obvious features for emotion recognition when humans listen to music (Hsu et al., 2017), and later there were also many efficient single-mode networks designed for this type of data (Lv et al., 2022; Ye et al., 2023; Ju et al., 2024; Wang et al., 2024). The 3D representation of EEG signals is used for learning 3D convolutional neural networks (Salama et al., 2018), thermal imaging of facial expressions is used for emotion recognition research (Gupta and Sengupta, 2023), and so on. Continuously studying emotion recognition methods from new perspectives is worthy of recognition. It fully utilizes neural networks to learn data features, and related research has also achieved certain results. In a word, all the above researches are based on the single mode method.

### 2.2 Emotional recognition in multi-modal settings

In order to fully utilize the features in the data and enhance the interactivity between data features, multi-modal fusion between data is particularly important. Recently, a large number of studies have used multi-modal fusion methods, some of which rely on the features extracted by neural networks (Rayatdoost et al., 2020; Tan et al., 2020; Zhang et al., 2022), while others have undergone data transformation without using the original data (Nakisa et al., 2020; ZENG et al., 2020; Dolmans et al., 2021). At the same time, there have been many advances in the study of modal interactivity, such as the interaction between acoustic information and natural language (Sakurai and Kosaka, 2021), modal interaction between audio and video (Chang and Skarbek, 2021), and the emotional recognition of gestures and facial expressions (Avula et al., 2022). In recent studies, features of human facial data, speech data, and EEG signals have been fused at the decision level through three branches, and very significant breakthroughs have been made in utilizing such data for multi-modal emotion recognition tasks (Pan et al., 2023). Faced with the difficulty of using physiological signals to solve research gap of emotional recognition, Parameterized hypercomplex neural networks (PHNN) is proposed as an emerging family of models which operate in a hypercomplex number domain (Zhang et al., 2021; Grassucci et al., 2022). In the study of using hypercomplex theory to solve multi-modal signal emotion recognition, the focus of data feature learning is on the parameterized hypercomplex multiplication (PHM) layer in the latter half of the network (Lopez et al., 2023).

One of the key steps to improve the effectiveness of multi-modal learning is the attention network, which can simulate human behavior to classify the information we obtain, filter secondary information, and grasp the main information. The use of attention can effectively enable the model to grasp the key parts of the many features in the data (Chen et al., 2020). Secondly, fusion strategy is also a key step that affects multi-modal networks. Early fusion methods did not consider the properties of different modalities, which can easily overlook the complementary information between modalities. Later fusion methods can easily cause network complexity, and more importantly, they cannot fully utilize cross modal information (Kaliciak et al., 2014; Gadzicki et al., 2020). A fusion strategy that combines the advantages of the above two methods, namely hybrid fusion, also known as intermediate fusion, is relatively complex and requires full consideration of various complexity issues (Stahlschmidt et al., 2022). In this paper, we propose a new network model based on the theory of hyper complex multi-modal emotion recognition networks. This network model can not only grasp the weight relationship of multiple modalities through attention, but also interact the fused modal information with the PHM layer in the decoder to obtain better modal interaction information.

## 3 Method

### 3.1 Framework overview

**Figure 2:**
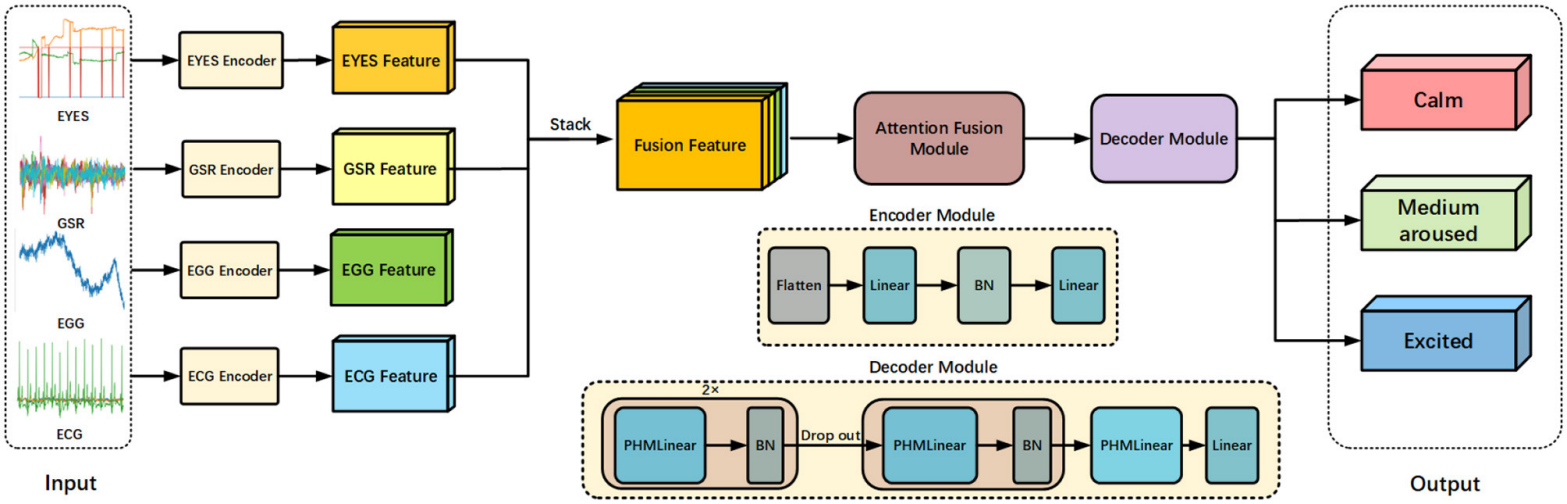
Overall framework of our proposed approach. We extract multi-modal features of signals through the following methods. The signals of four modes are used as inputs and converted into features for fusion by their respective encoders. The architecture of the encoders includes a linear layer and a batch normalization layer. The main features is emphasized in our designed attention fusion module. The fused features of the final signal are refined by a decoder containing a PHM layer, and ultimately mapped to the output.

In Figure 2, we present a streamlined and efficient architecture for emotion recognition of multi-modal signals, utilizing a novel fusion approach and attention mechanisms. This architecture, termed the multi-modal hyper complex fusion network, optimizes the integration of modal information from various physiological signals to enhance sentiment classification accuracy. The framework comprises three main components: (i) a data encoder, which extracts features from the modal data of each signal while converting its represented feature dimensions into consistent ones through linear layers. (ii) Attention fusion module, which aims to multiply the fused multi-modal data with the weight matrix. Through continuous training of the model, the final model will focus on the main features when processing information. (iii) Feature decoder, which decodes the output from the attention fusion module and further transforming dimensions through the PHM layer and linear layer for comprehensive sentiment recognition of multi-modal signal data.

Firstly, we extract features from the data of four physiological signals using an encoder, comprising linear mapping and normalization operations. Among these, the EEG, ECG, and EYE encoders traverse the data through the linear layer and batch normalization layer within the module twice, whereas GSR only requires a single pass. This encoder standardizes the feature dimensions of all four modalities to 512, followed by feature-level fusion to create features of 8 × 4 × 512 dimensions. Secondly, we employ five attention modules to focus on the fused main features across the four channels, assigning weights to modal features from different channels. This module is collectively referred to as the attention fusion module within the model. Finally, we utilize the PHM layer in the feature decoder to capture both local and global information from the fused features, and a linear layer to map the weighted features into dimensions, culminating in the downstream task of sentiment recognition.

### 3.2 Input and output modeling

Our model begins with four distinct dimensions of signal modal data, all comprising waveform data. In the preprocessing stage prior to model input, we adopted processing methods from prior studies on this dataset. Specifically, for EEG, ECG, and GSR signals, we conducted downsampling operations, reducing their frequencies from 256Hz to 128Hz. Conversely, for eye data, we retained its original frequency and applied relevant filters. Thus, the dimensions of the input data are as follows: EYES [8,600,4], GSR [8, 1,280], EEG [8, 1,280, 10], and ECG [8, 1280, 3]. By utilizing encoders tailored to each of the four signal modes, we standardized the signals into unified [8, 512] dimensional features, ensuring suitable data input for the attention fusion module.

Moreover, we preserved the linear mapping aspect of the PHM layer from the original hyper-complex multi-modal fusion model to apply to the fused features. This ensures that our model's decoder maintains the original mapping relationship with the output of the attention fusion module.

### 3.3 Early interaction

In previous research on hyper-complex multi-modal fusion networks, these four modal data types were fused via batch alignment. However, only the PHM layer could map the feature vectors of the four modalities in the fused data, which did not effectively capture the impact of each modality on final emotion recognition. To address this, we employed modal fusion by stacking. The fused modal features were then processed through our designed attention module, which includes five types of attention based on the fused features. We conducted experiments using these attention mechanisms, which were developed from channel attention, self-attention, and enhanced modal frameworks.

With our proposed fusion method and attention model, each modality can be assigned its respective weight before entering the PHM layer. Finally, guided by the decoder, emotion recognition is performed, establishing a novel multi-modal emotion recognition network. Additionally, we adjusted the early stopping parameter based on the model's adaptation to various attention levels to mitigate potential overfitting issues.

### 3.4 Attention fusion module

The attention fusion module we designed encompasses five types of attention tailored for multi-modal physiological signal research. Each type of attention network is individually used as our attention fusion module to realize its own function in the network. Drawing inspiration from channel attention, self-attention, and their variant frameworks, our aim is to enhance the training performance of hyper-complex multi-modal neural network models. The following is an in-depth explanation of our proposed Species Attention Fusion Module, including the framework for modeling and the underlying principles of the formulas.

#### 3.4.1 Signal channel squeeze and excitation attention

**Figure 3:**
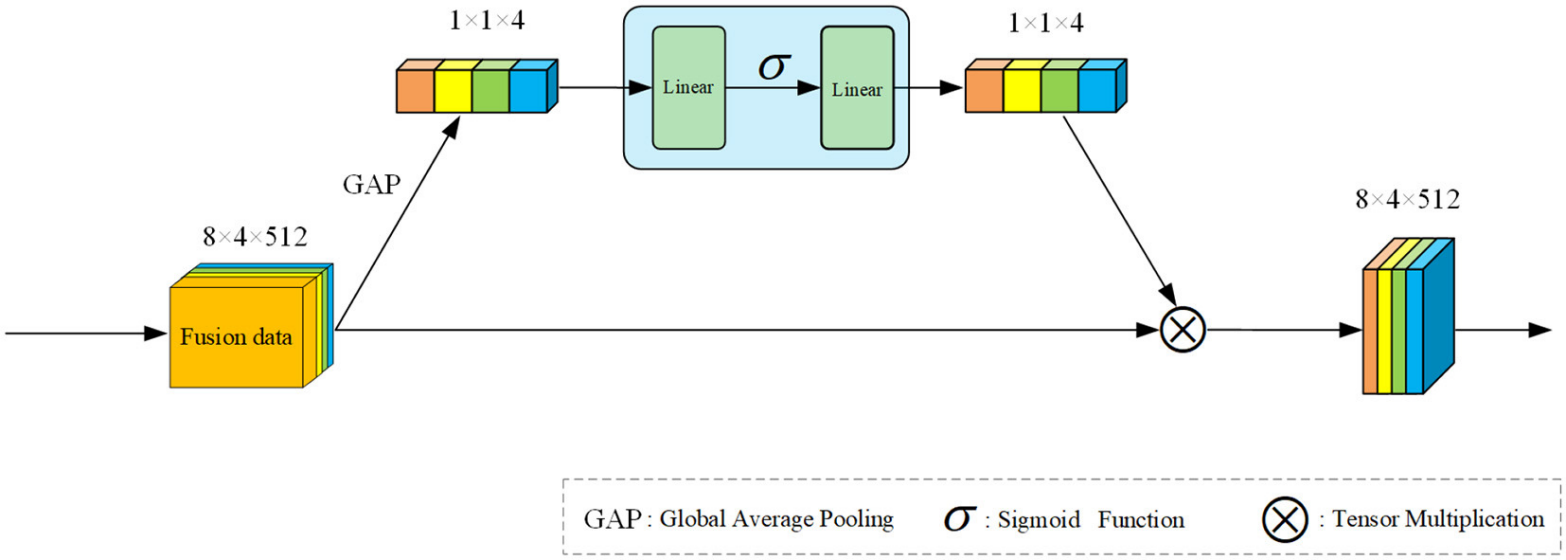
Overall framework of our proposed SCSEA. The input feature data is channel-level extracted through Global Average Pooling (GAP). Squeezing and extraction are performed in two separate linear layers, with non-linear activation applied using an activation function. The resulting attention weight matrix is then multiplied with the original feature data.

We drew inspiration from the block design in SE net (Hu et al., 2018) and identified that extracting channel features for modal weighting could enhance training performance. Consequently, we devised the Signal Channel Squeeze and Excitation Attention (SCSEA) module, comprising three pivotal steps. The module's framework diagram is illustrated in Figure 3. The initial step maximizes input feature pooling to derive channel features, followed by channel feature extraction via linear layers in the second step. Finally, the third step involves channel feature extraction from functions processed by activation functions.

Assuming *x* is the input signal fusion feature. *x* ∈ *R*^B*C*F^. Where B represents batch size, C represents channel, and F represents the characteristics of the channel. After performing global average pooling, the features of *x* are preliminarily extracted now *x* ∈ *R*^1*1*C*1^. Before that, there was actually a dimension extension operation that changed $x_{0}\in R^{B*C*F*1}$ to $x_{1}\in R^{1*1*C*1}$. Then, the feature is further transformed into $x_{2}\in R^{1*1*C/r*1}$ with the aim of having the activation function act on it and then restore it to the same dimension as *x*_1_ through a linear layer. We assume it as *x*_3_, and the entire process of this attention can be expressed by the following formula:


(1) $$\begin{array}{l} x_{1}=GAP\left(x_{0}\right) \end{array}$$

(2) $$\begin{array}{l} y=f\left(\sigma \left(f\left(x_{1}\right)\right)\right)*x_{0} \end{array}$$

where *GAP* is the global average pooling operation, σ is the activation function, and *f* is the linear mapping function.

Using the data from our research as an example, the fusion features of the four signals need to undergo dimension expansion from [8, 4, 512] to [8, 4,512, 1] before entering the attention module. This expansion aims to allow the attention module to adjust the weight of the channel dimension. Initially, the input data is represented as channel features via a max-pooling layer, altering the data dimension to [1, 1, 4, 1]. Here, each modality's data is compressed into a single dimension, making its scattered features more accessible. Next, a linear layer squeezing operation extracts the four most prominent channel features, followed by activation with a ReLU function for nonlinear mapping. Subsequently, the dimension of the weight matrix obtained from this extraction changes to [1, 1, 4, 1], and it multiplies with the original input fusion features to fulfill the attention module's final objective. Lastly, a summation in the channel dimension is performed to assist the encoder in decoding the features.

#### 3.4.2 Efficient signal channel attention

**Figure 4:**
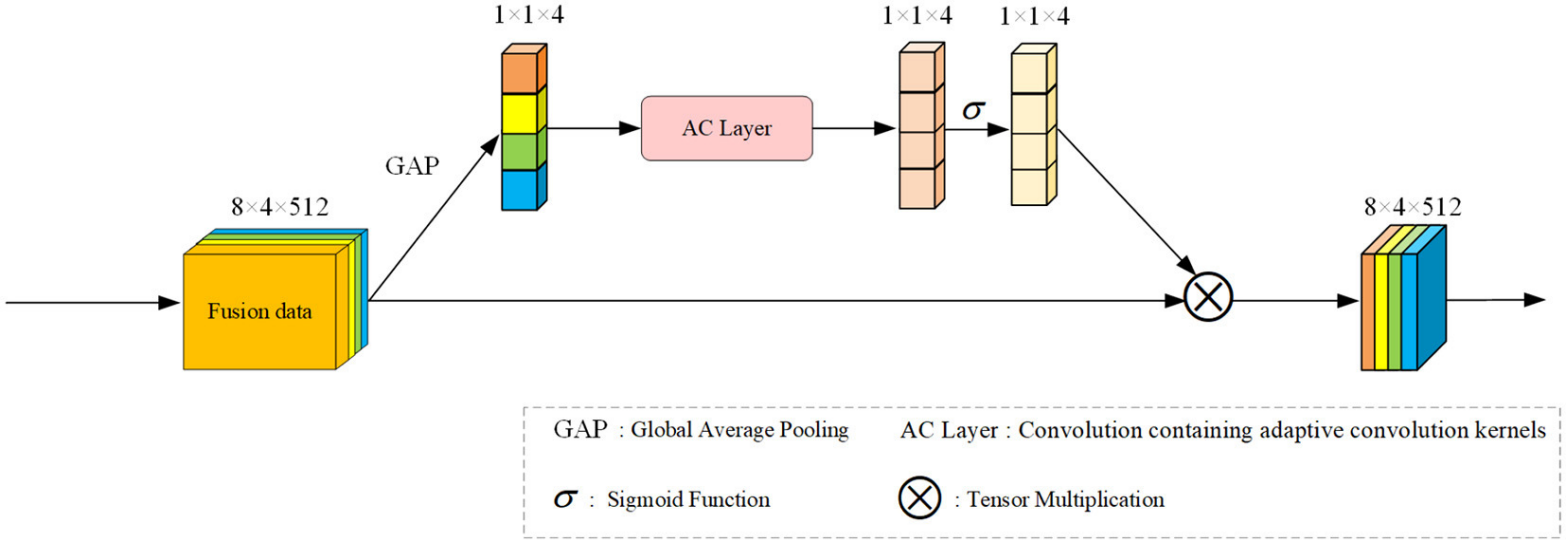
Overall framework of our proposed ESCA. We'll extract channel features from the input feature data using the GAP method. This method conducts convolutional extraction of local features within the channel in our designed AC Layer, resulting in a weight matrix under the activation function's influence. This tensor is then multiplied with the original input to generate our output.

Inspired by EC Attention (Wang et al., 2020), our approach for signal fusion features calculates the attention matrix from a different perspective, focusing on the convolution angle. For sequential data like signals, an appropriate convolutional kernel proves more effective. Building upon the methods and principles of EC Attention, we've devised an attention module tailored for our fusion signal, termed Efficient Signal Channel Attention (ESCA). Refer to Figure 4 for the module's framework diagram.

Given our input feature *x*_0_, similar to the previous SCSEA, we perform an average pooling operation to preliminarily extract channel features, resulting in *x*_1_. Then, through a linear mapping, it is transformed into a dimension that can interact with the convolution kernel, and then subjected to one-dimensional convolution. We have designed a convolution layer for *x*_1_, called AC Layer, which includes using appropriate convolution kernels for convolution operations. After performing one-dimensional convolution, we obtained result *x*_2_ and obtained the weight matrix of attention through non-linear mapping using an activation function. By multiplying it with the original input features, we obtained the purpose of our attention. This process can be expressed as a function:


(3) $$\begin{array}{l} y=\sigma \left(f_{AC}\left(GAP\left(x_{0}\right)\right)\right)*x_{0} \end{array}$$

where *GAP* is the global average pooling operation, Σ is the activation function, and *f*_*AC*_ is the adaptive convolution function.

#### 3.4.3 Signal feature dot product block

Both of the approaches mentioned above involve attention calculation at the channel level. Building on the principles of SDP Attention (Vaswani et al., 2017), we've revamped our strategy by integrating *Q*, *K*, and *V* as input matrices for the attention function, dubbing it Signal Feature Dot Product Block (SFDPB).

**Figure 5:**
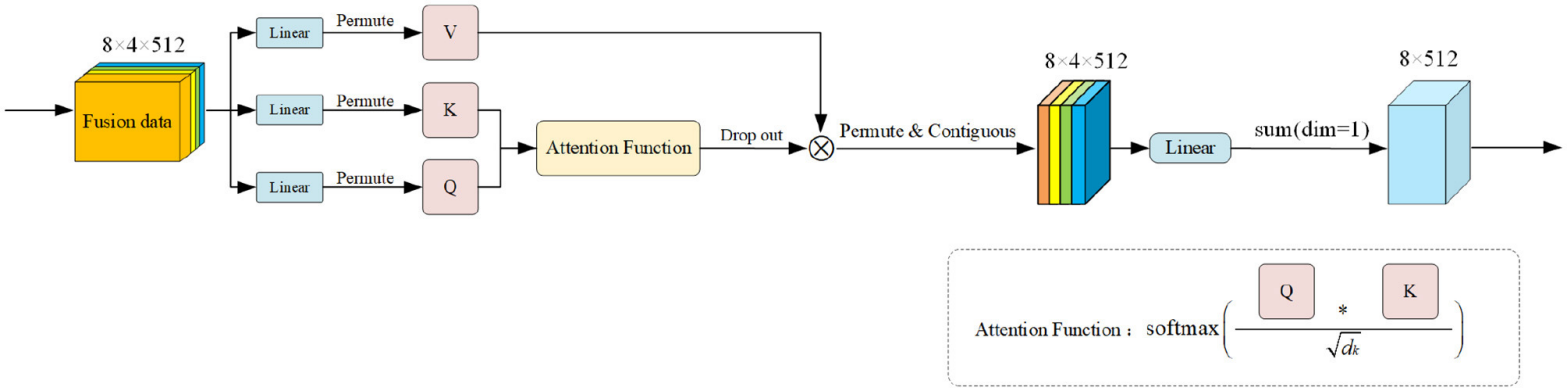
Overall framework of our proposed SFDPB.The input feature data undergoes linear mapping across three designated linear layers to derive *Q*, *K*, and *V* matrices. *Q* and *K* undergo operations and dropout within specific attention functions to bolster feature representation. The resultant matrix is then multiplied by the *V* matrix, and the data is permuted and concatenated to restore the feature dimension. Lastly, we sum up the dimensions with *dim* = 1 to yield the output result.

Refer to Figure 5 for the module's framework diagram. This shift stems from our team's recognition of the benefits of self-attention, particularly in capturing global features and showcasing exceptional adaptability. Given the sequential nature of signal data, besides global features, capturing its inherent characteristics poses a significant challenge. Moreover, the psychological signal data we examine also displays substantial volatility, necessitating attention functions with robust adaptability. The calculation method of this attention function is:


(4) $$\begin{array}{l} f\left(Q,K,V\right)=V*softmax\left(\frac{QK^{T}}{{\sqrt{d}}_{k}}\right) \end{array}$$

where *Q* is the query matrix, *K* is the key matrix, and *V* is the value matrix. *d*_*k*_ is the dimension of the key matrix. We have maintained the same approach as the original author in calculating this attention function, as this method has been proven to be more effective. Even with the use of previous methods, we have fine tuned the internal attention of the signal fusion feature format in this study to make it effective in the network we are studying. For example, we set the *d*_*v*_, *d*_*k*_, and *h* parameters during the calculation process to 64, 64, and 8, respectively, and we add and sum the dim=1 dimension of the attention calculation result to facilitate addition at the decoder level.

#### 3.4.4 Signal attention free block

**Figure 6:**
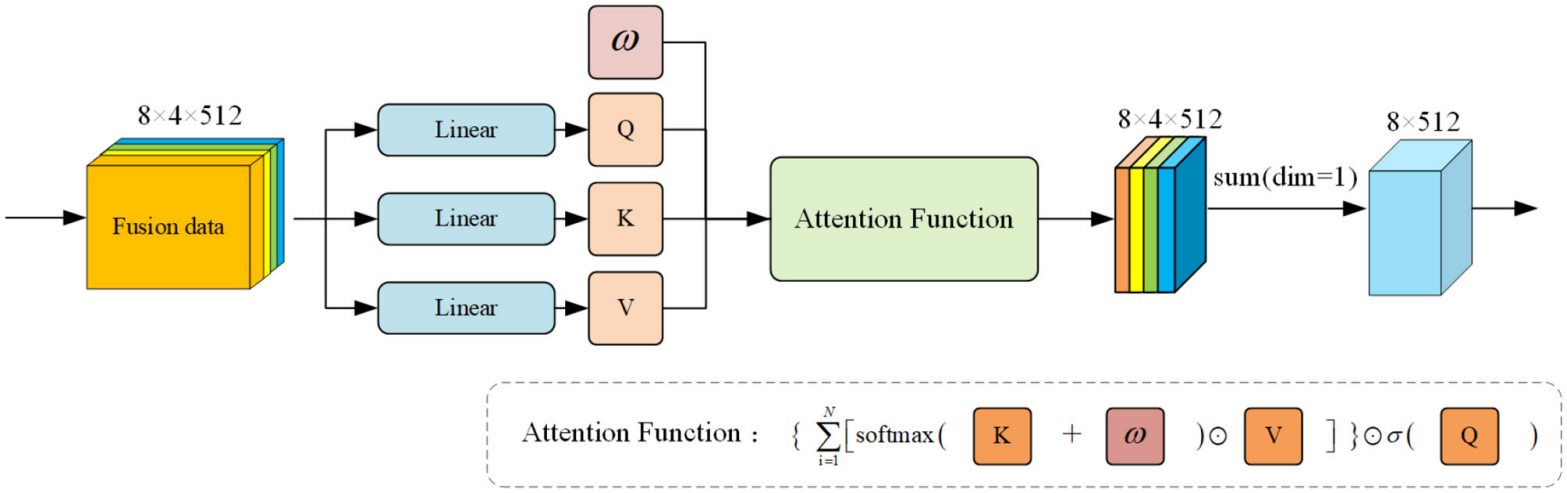
Overall framework of our proposed SAFB. The input feature data is mapped linearly across three designated linear layers to derive *Q*, *K*, and *V* matrices. Initially, a weight is initialized and operated on with *Q*, *K*, and *V* within specific attention functions to obtain preliminary weighted features. To aid the decoder's final linear mapping, we then sum the dimensions with *dim* = 1 to produce the output result.

Following the previous calculation approach, the matrices *Q*, *K*, and *V* are derived through linear transformation of the original data features. In contrast to SDP attention, AFT attention employs a different attention function calculation method (Zhai et al., 2021). Inspired by the structure of AFT attention, we devised our Signal Attention Free Block (SAFB), as depicted in Figure 6.

For data signals, each feature within a single channel undergoes a weighted average of AFT execution values, which are then combined with element-wise multiplication queries. This attention calculation method simplifies the weight computation, relying solely on a key and a set of learned paired positional deviations, offering the direct advantage of avoiding the need to compute and store costly attention matrices. We also drew on its advantages to produce this attention block that resonates with the model we are studying. The calculation of this attention function is as follows:


(5) $$\begin{array}{l} y_{output}=\sigma \left(Q\right)⊙\overset{N}{\underset{i=1}{\sum }}\left[softmax\left(Q+w\right)⊙V\right] \end{array}$$

where *Q* is the query matrix, *K* is the key matrix, and *V* is the value matrix. *d*_*k*_ is the dimension of the key matrix. ⊙ represents the product of two elements before and after and *w* is the weight matrix generated by initialization.

#### 3.4.5 Multiscale signal transformer block

**Figure 7:**
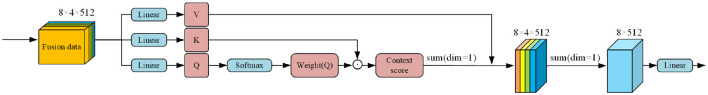
Overall framework of our proposed MSTB. The input feature data undergoes linear mapping across three specific linear layers to derive *Q*, *K*, and *V* matrices. The *Q* matrix requires non-linear mapping through an activation function to obtain its corresponding weight matrix. This weight matrix is then multiplied with the *K* matrix to obtain the context score matrix. Summation along *dim* = 1 is performed to reduce feature space occupation, followed by multiplication with the *V* matrix to yield preliminary results. Similarly, akin to the previous module, dimension summation with *dim* = 1 occurs, followed by linear layer mapping to obtain the final result.

When we don't focus on processing signal sequence data, attention in the field of computer vision will also achieve good results. MVITv2attention is an example of using matrices *Q*, *K*, and *V* (Li et al., 2022). The way we obtain these three matrices in our designed attention remains consistent with the previous text, but it reduces pooling operations compared to MVITv2attention, so we will not elaborate on it here. We found that MVITv2 attention changed the calculation method of the *Q*, *K*, and *V* matrices. Our research retained some of its advantages in the module and designed a new attention, which is Multiscale Signal Transformer Block (MSTB). Its framework diagram is shown in Figure 7.

The expression for this attention is as follows:


(6) $$\begin{array}{l} y_{c}=K⊙softmax\left(I\right) \end{array}$$

(7) $$\begin{array}{l} y_{output}=f_{dsum}\left(f_{dsum}\left(y_{c}\right)⊙V\right) \end{array}$$

where *Q* is the query matrix, *K* is the key matrix, and *V* is the value matrix. *y*_*c*_ represents context score, which is an intermediate variable for us to calculate the weight matrix. ⊙ represents the product of two elements before and after and *w* is the weight matrix generated by initialization. *y*_*c*_ is a function that sums up a specific dimension. In this module, we default to summing up in the *sum* = 1 dimension. The main function of MSTB is to capture the contextual features of each modality, sum them up in specific dimensions, and reduce the space occupation of features, which is more conducive to the decoder's feature classification.

## 4 Experiments

### 4.1 Dataset and evaluation metrics

#### 4.1.1 Dataset

All experiments in this study were conducted using the publicly available MAHNOB-HCI dataset, consistent with the original research on hypercomplex multi-modal emotion recognition networks. This dataset encompasses diverse physiological response data alongside subjective emotional reports from participants. Specifically, it serves as a multi-modal resource for emotion recognition, comprising synchronized recordings of facial videos, audio signals, eye gaze data, and peripheral/central nervous system physiological signals from 27 participants viewing emotional video clips. Eye gaze data includes attributes like eye distance, pupil size, and gaze coordinates. For our physiological signal recognition study, we focused solely on EEG, ECG, and GSR due to their strong correlation with human emotions. Additionally, the dataset provides relevant labels including calm, moderate arousal, excitement, and valence categories (unpleasant, neutral, and pleasant). However, within our multi-modal model, our emphasis was on validating method effectiveness, thus concentrating on emotion recognition for three specific labels: Calm, Medium Aroused, and Excited. The whole dataset was processed through data processing on the basis of the original MAHNOB-HCI dataset, 80% of the data was constructed as a training set, and 20% of the data was constructed as a test set. The divided training and test sets are then put into the model for training.

#### 4.1.2 Evaluation

We utilize accuracy and F1 score as evaluation metrics for our model. Accuracy indicates the percentage of correct predictions made by the model across the entire sample, while the F1 score, being the harmonic mean of accuracy and recall, offers a balanced assessment of model prediction performance. The F1 score can be calculated using the following formula:


(8) $$\begin{array}{l} Pr=\frac{TP}{TP+FP} \end{array}$$

(9) $$\begin{array}{l} Re=\frac{TP}{TP+FN} \end{array}$$

(10) $$\begin{array}{l} F1=2*\frac{Pr*Re}{Pr+Re} \end{array}$$

Where *TP* (True Positive) is the number of true positive classes that were correctly predicted, *FP* (False Positive) is the false positive class prediction, and *FN* (False Negative) is the false negative class prediction. *Pr* and *Re* represent precision and recall.

These metrics together provide a comprehensive evaluation of the model quality. Moreover, to address overfitting concerns, various early stopping parameters were employed for different attention modules in our study. Ablation experiments were conducted using the attention module that exhibited the best classification performance.

### 4.2 Implementation details

#### 4.2.1 Architecture

The input signal comprises four modes: EYES with dimensions [8, 600, 4], GSR with dimensions [8, 1,280], EEG with dimensions [8, 1,280, 10], and ECG with dimensions [8, 1,280, 3]. Utilizing encoders corresponding to each signal mode, we convert these signals into unified features of dimensions [8, 512]. In the feature fusion module, we merge features of the same dimension at the feature level before inputting them into the attention fusion module. Our study employs five distinct types of attention fusion modules, each with its unique structure. The decoder consists of multiple *n* = 4 PHM layers and intersecting normalization layers. In each PHM layer and normalization layer within the decoder, the feature dimension is halved, culminating in the final prediction output.

#### 4.2.2 Training

In our model, we employed the Adam optimizer with a fixed learning rate of 0.000000796 and zero weight decay. Training was conducted on a single Nvidia RTX4090 GPU, utilizing a total batch size of 8, with all networks operating on this GPU. Optimization was achieved using CrossEntropy Loss, aligning predicted physiological signal labels with actual emotional categories.

### 4.3 Comparison with previous works

From the results of this experiment, we observed variations in replicating the original hypercomplex neural network model's results. Further analysis unveiled missing and erroneous data within our study. Consequently, we purged these data points, obtaining a clean dataset suitable for training, albeit influencing our final model predictions. Notably, the original paper didn't address this issue or offer solutions based on their findings. We reproduced the initial hypercomplex neural network model (Lopez et al., 2023), which is named “Baseline” in our experiment. The most notable differences from our study are the modal fusion approach and the lack of an attentional module. Our experiments encompassed both unaugment and augment datasets, with the comparative results detailed in Table 1.

**Table 1:** The table illustrates the performance of each attention module with augment data, with model evaluation metrics categorized into accuracy and F1 score.

**Attnetion module**	**Early stopping**	**Accuracy**	**F1 score**	**Parameters**
Baseline	20	34.4482	0.3196	19663747
ESCA	20	42.4749	0.3251	18506051
SFDPB	20	40.1338	0.1909	17913091
SAFB	8	38.4615	0.2308	17913091
MSTB	20	40.1338	0.1909	17913091
SCSEA	8	**43.1438**	**0.3328**	17847451

“Early stopping” represents the number of training iterations needed for the model when the training loss shows signs of increase. “Parameters” represents the number of parameters included in the model. The bold values means that the corresponding model has the best performance for the corresponding metrics.

Utilizing five types of attention, the highly intricate multi-modal physiological signal sentiment classification model demonstrated notable enhancements, particularly with SCSEA and ESCA, resulting in substantial overall improvements in accuracy and F1 score. Additionally, the five attention modules we developed each contributed to a reduction in the model's parameter size to varying extents, with the SCSEA module demonstrating the most substantial decrease. Furthermore, as indicated in Table 1, the attention modules SFDPB, SAFB, and MSTB exerted a comparable impact on the model's parameter size, a result of their similar design principles. Although they differ slightly in computation methodology, the specifics can be referenced in the module framework diagram provided above. While the remaining three attention types exhibited accuracy improvements, their F1 scores notably declined. The reason for this phenomenon should be the following two reasons:

1) When dealing with a modest amount of data, certain discrepancies become more pronounced. This is particularly true for the F1 score calculation, which includes inverse operations, making the disparities in predictive balance particularly evident.
2) The F1 score serves as a holistic measure of both precision and recall. Incorporating the three sub-attention modules leads to a reduction in recall, consequently boosting the accuracy, which inversely affects the F1 score, causing it to decline.

Consequently, integrating these three attention types enhanced the model's prediction accuracy, albeit resulting in a more unbalanced prediction compared to the other two attention types.

### 4.4 Ablation study

From the preceding analysis, it's evident that SCSEA experienced the most significant improvement post-model addition. We conducted ablation experiments on multi-modal physiological signals, comparing single-mode (GSR signal), dual-mode (GSR and ECG signals), three-mode (GSR, EEG, and ECG signals), and four-mode (EYES, GSR, EEG, and ECG signals) scenarios. The Table 2 showcases the experimental results under consistent experimental conditions and parameters.

**Table 2:** Results table of modal ablation experiment.

	**Modal**				**Results**	
	**EYES**	**GSR**	**ECG**	**EGG**	**Accuracy**	**F1 score**
Exp1	-	+	-	-	37.4581	0.3299
Exp2	-	+	+	-	33.1104	0.3206
Exp3	-	+	+	+	41.1371	0.2972
Exp4	+	+	+	+	**43.1438**	**0.3328**

The “+” represents the use of the corresponding modality, while the “-” corresponds to the removal of the corresponding modality. The bold values means that the corresponding model has the best performance for the corresponding metrics.

The results of the ablation experiment clearly indicate the necessity of modal synergy. Despite poorer dual-modals performance in the “exp2” experiment, the overall trend highlights that leveraging interaction among the four modalities is the most effective approach for improving emotion signal recognition. Compared to initial single-mode results, interaction among the four physiological signals yielded superior accuracy and F1 scores. Boosted by our optimal attention module from “exp4,” accuracy improved by approximately 6% compared to single-mode experiments, while the F1 score rose by 0.004. However, significant enhancement of the F1 score remains limited by data diversity. Despite efforts to enrich existing data, this limitation persists.

### 4.5 Discussion

Our proposed SCA-Net offers three key advantages:

1) It integrates the features of the four modalities-EEG, ECG, GSR, and EYE-along the channel dimension, enabling the attention fusion module to assign weights directly to each modality. This allows the primary modality to take precedence while secondary modalities contribute differently to the final classification task in emotion recognition.
2) We have validated multiple attention methods and demonstrated that channel attention is more appropriate for our network design than self-attention, as evidenced by its superior accuracy, F1 score, and parameter count. Specifically, the SCSEA module we developed compresses and extracts features from individual channels, providing a more direct response to the core characteristics of each modality. As a result, SCSEA outperforms all other attention modules.
3) For the output of the attention fusion module, we have adapted its overall dimensionality to align with the features of the PHM layer, which in turn facilitates the PHM's decoding and pattern recognition tasks on the weighted data features.

These three complementary advantages collectively enhance the performance of SCA-Net, surpassing that of the original hypercomplex multi-modal neural network model in the realm of emotion recognition using multichannel physiological signals.

## 5 Conclusion

In this study, we introduce five novel attention-based hypercomplex models for sentiment recognition of physiological signals. After conducting our experimental research, we've found that SCA-net, as a multi-modal neural network, exhibits the most significant enhancement in model performance. These signal models are trained on data from four physiological signals. By incorporating an attention layer into the hypercomplex layer, which already captures feature relationships, each modality is appropriately weighted before entering the linear layer, effectively enhancing model predictive performance. However, we observed that while partial attention improves accuracy, it doesn't ensure balanced predictions. Additionally, even in attention networks with strong predictive performance, there's room to improve F1 scores. Thus, achieving a balanced prediction in the hypercomplex physiological signal emotion classification model represents a significant research milestone. In future research, we will aim to streamline the model parameters and refine its structure, ensuring concurrent enhancements in both accuracy and F1 score. In addition, we would like our proposed attention module to be utilized in more advanced fusion networks, such as Fuzzy-Based Deep Attributed Graph Clustering (Yang et al., 2023b), in order to facilitate the accuracy of the corresponding models. The novel network architecture we proposed in our study also has the potential to be used in the field of RNA N6-methyladenosine modification site prediction and drug repositioning in the future after our improvement and refinement (Li et al., 2022, 2024).

## Data availability statement

Publicly available datasets were analyzed in this study. This data can be found here: https://mahnob-db.eu/hci-tagging/.

## Author contributions

ZD: Data curation, Formal analysis, Validation, Writing – original draft, Writing – review & editing. YX: Conceptualization, Funding acquisition, Project administration, Writing – review & editing. PZ: Supervision, Writing – review & editing.
